# An Optimized Approach to Recover Secreted Proteins from Fibroblast Conditioned-Media for Secretomic Analysis

**DOI:** 10.3389/fncel.2016.00070

**Published:** 2016-03-31

**Authors:** Bastien Paré, Lydia T. Deschênes, Roxane Pouliot, Nicolas Dupré, Francois Gros-Louis

**Affiliations:** ^1^Division of Regenerative Medicine, Laval University Experimental Organogenesis Research Center/LOEX, CHU de Québec Research Center – Enfant-Jésus HospitalQuébec, QC, Canada; ^2^Department of Surgery, Faculty of Medicine, Laval UniversityQuébec, QC, Canada; ^3^Faculty of Pharmacy, Laval UniversityQuébec, QC, Canada; ^4^Neuroscience Division of the CHU de Québec, Department of Medicine of the Faculty of Medicine, Laval UniversityQuébec, QC, Canada

**Keywords:** secretomic, 2D-DIGE, secreted proteins, secretome, fibrolast-conditioned media

## Abstract

The proteins secreted by a particular type of cell, the secretome, play important roles in the regulation of many physiological processes via paracrine/autocrine mechanisms, and they are of increasing interest to help understanding rare diseases and to identify potential biomarkers and therapeutic targets. To facilitate ongoing research involving secreted proteins, we revisited cell culture protocols and whole secreted protein enrichment protocols. A reliable method for culturing and precipitating secreted protein from patient-derived fibroblast conditioned-medium was established. The method is based on the optimization of cell confluency and incubation time conditions. The well-established carrier-based TCA-DOC protein precipitation method was consistently found to give higher protein recovery yield. According to our results, we therefore propose that protein enrichment should be performed by TCA-DOC precipitation method after 48 h at 95% of confluence in a serum-deprived culture medium. Given the importance of secreted proteins as a source to elucidate the pathogenesis of rare diseases, especially neurological disorders, this approach may help to discover novel candidate biomarkers with potential clinical significance.

## Introduction

The secretome, a complex set of molecules secreted from living cells, represents a major class of proteins. It is considered to be encoded by ~10% of the human genome (Pavlou and Diamandis, 2010). It is well known that secreted proteins are associated with a broad range of physiological processes and play a key role in homeostasis, growth, differentiation, proteolysis, apoptosis, signaling, cell adhesion, and development of the extracellular matrix (ECM; Luo et al., 2012; Stastna and Van Eyk, 2012). Furthermore, the proteins secreted by particular types of cells are of increasing interest as a potential source of biomarker discoveries and therapeutic targets in diseases. Although the proteins released by cells into conditioned-media *in vitro* have been studied abundantly to better understand pathological conditions and mechanisms, the concept of the secretome has become an attractive and challenging proteomic technology in recent years.

There has been an increasing number of studies over the last decade targeting the secretome characterization, spanning a wide range of biological samples from microorganisms and lower animal models to human embryos and cell lines leading to the generation of a valuable secretome catalogs (Leroy et al., 2006; Klee, 2008; Katz-Jaffe et al., 2009; Brown et al., 2013; Gelman et al., 2013; Hartwig et al., 2013; Makridakis et al., 2013; Meissner et al., 2013). These efforts have been greatly facilitated by significant technological advances in the field of proteomics during the last two decades [improvement of proteomic platforms, mass spectrometry instrumentation and 2D-difference gel electrophoresis (2D-DIGE); Klose, 1975; O'Farrell, 1975; Beckett, 2012; Mukherjee and Mani, 2013]. The 2D-GE technique, introduced in 1976, enables protein separation according to their isoelectric point (pI) and molecular weight (Bensadoun and Weinstein, 1976; Peterson, 1977; Feist and Hummon, 2015). The 2D-DIGE technique addresses the problems encountered in the 2D-GE technique, which were a lack of reproducibility and quantification, and was introduced by Unlü et al. (Unlü et al., 1997). Using CyDyes (2, 3, and 5) for protein labeling, which are fluorescent dyes, the samples can be pooled and separated on one single 2D-PAGE (polyacrylamide gel electrophoresis) gel and then scanned by detecting each CyDye independently. The spots observed can be analyzed for expression levels and extracted from the gels for further analysis by mass spectrometry. The direct advantages of this technique is the use of an internal standard, generally a protein pool conjugated with the Cy2 fluorescent dye, and the multiplexing of the samples implying the simultaneous measurement of multiple analytes in a single analytical run and therefore increasing the power for statistical analysis (Minden, 2007). Along these lines, there has been a growing effort over the last few years to analyze fibroblast secretomes by proteomic approaches (secretomics), to allow the identification of molecules secreted by fibroblast cells and consequently gain insight into the mechanisms of immunomodulation, inflammation, angiogenesis, cell survival, or cell differentiation and recruitment in various health and diseases or conditions. Despite these advances, secretome analysis still faces some difficulties mainly related to sample collection and preparation.

The secretome is a challenging sample to analyze, mainly due to inherent difficulties with sample collection and preparation since secreted proteins are quite diluted and often present in μg to ηg. In addition, culture media often contains salts and other compounds such as phospholipids, lipids and polysaccharides, which interfere with most proteomics techniques. Thus, selective carrier-based precipitation of proteins becomes almost mandatory for proper subsequent secretomic analysis (Chevallet et al., 2007). Therefore, since the analysis of secreted proteins is challenging due to technical difficulties related to cell collection and preparation, the principal aim of this study was to determine the optimal time to harvest fibroblast conditioned-media and the best protein precipitation and conjugation methods for the subsequent secretomic screening experiments. A variety of methods have been previously described to extract diluted secreted proteins usually present in conditioned culture media in low concentration. These techniques present several drawbacks, such as coprecipitation of salts or poor yields at low protein concentrations. Samples should have a high protein concentration and be free of salt and other disturbing factors, such as ionic detergents, nucleic acids, and lipids that could interfere with downstream analysis. Protein concentration methods, such as absorption to hydrophobic resin, ultrafiltration, lyophilisation, and dialysis, are therefore not ideal for subsequent secrotomic analysis. From the data already present in the literature, we ruled out two carrier-based protein precipitation methods proven to be highly efficient to recover diluted proteins from culture media, trichloroacetic—sodium deoxycholate (TCA-DOC) and tetrahydrofuran—sodium lauroyl sarcosinate (TCA-NLS-THF) (Bensadoun and Weinstein, 1976; Chevallet et al., 2007; Feist and Hummon, 2015). In the current work, we found that the TCA precipitation protocol we described, enabling protein precipitation from large sample volumes, gave high yield protein recovery.

## Materials and methods

### Sample selection and collection

All cases signed a consent form approved by our Institutional Ethics Committees prior to being enrolled in the study and were recruited on a voluntary basis (Ethical research board of the CHU de Québec. Protocol number: 2012-1316. For more information, please contact gurecherche@chuq.qc.ca). Skin biopsies were collected from affected and unaffected individuals. Two different pathologies and control individuals were targeted in order to fully validate our results. Besides collecting skin biopsies, blood samples were also collected and used for DNA extraction and patient's genotyping to confirm diagnosis when possible. In total, 6 Amyotrophic Lateral Sclerosis (ALS), 3 neurofibromatosis type 1 (NF1) cases, and 3 controls individuals were recruited for this study (Table 1). Fibroblasts were used in this study as they are known to be affected in different neurological conditions as ALS and NF1 (De Schepper et al., 2005; Maertens et al., 2007; Paré et al., 2015; García-Romero et al., 2016).

**Table 1:** **Detailed clinical information of patients and controls enrolled in this study**.

**ID number**	**Biopsy location**	**Sex**	**Age at sampling**	**Current age**	**Age of death**	**Clinical status**	**Genetic status**
SALS 1	Arm	M	48	NA	NA	Affected	No known ALS-associated mutations
SALS 2	Arm	M	62	NA	NA	Affected	No known ALS-associated mutations
SALS 3	Arm	M	81	NA	NA	Affected	No known ALS-associated mutations
FALS1	Arm	F	63	65	NA	Clinically unaffected	*C9orf72* expansion
FALS2	Arm	F	47	49	NA	Clinically unaffected	*C9orf72* expansion
FALS3	Arm	F	59	60	NA	Affected	*C9orf72* expansion
NFM1	Arm	M	25	26	NA	Affected	NA
NFM2	Arm	M	26	27	NA	Affected	NA
NFM3	Arm	F	22	23	NA	Affected	NA
CTRL 1	Arm	F	68	69	NA	Control	NA
CTRL 2	Arm	M	23	39	NA	Control	NA
CTRL 3	Arm	M	29	45	NA	Control	NA

*SALS, Sporadic ALS; FALS, Familial ALS; NFM, Neurofibromatosis type 1; CTRL, Control; M, Male; F, Female; NA, not applicable or info not available*.

### Skin biopsies and fibroblast cell extraction/culture

For each participant, two skin biopsies were collected using a 6-mm diameter punch biopsy. All skin biopsies were taken from the same body area for each subject and fibroblasts were isolated as described before (Paré et al., 2015). Briefly, the skin biopsies were incubated with 0.05% thermolysin (Sigma, Oakville, QC, Canada), overnight at 4°C, in order to facilitate the mechanical separation of the epidermis from the dermis. Then, fibroblasts were isolated from the dermis after treatment with 0.2 IU/ml collagenase H (Roche, Missisauga, Ontario, Canada). Cultured fibroblasts, grown in DMEM (Dulbecco-Vogt modification of Eagle's medium; Invitrogen, Burlington, ON, Canada) supplemented with 10% Bovine Growth Serum (HyClone BGS; GE Healthcare Life Sciences, HyClone Laboratories, Logan, UT, USA), 100 IU/ml penicillin G (Sigma, Oakville, QC, Canada), and 25 μg/ml gentamicin (Schering, Pointe-Claire, QC, Canada) in 5% CO_2_ at 37°C, at passage two were seeded at a concentration of 2.5 × 10^5^ cells on tissue culture dishes (75 cm^2^).

### Fibroblast induction and protein precipitation

Secreted proteins are often masked by high amounts of protein supplements in the culture medium. We have therefore developed a serum-deprived efficient method for the enrichment and analysis of the secretome of different fibroblast cell lines to overcome this difficulty. After three washes with 10 ml of phosphate-buffered saline (PBS) [0.8% (W/V) NaCl (BioBasic Inc., Markham, Ontario, Canada), 0.02% (W/V) KCl (BioBasic Inc., Markham, Ontario, Canada), 0.15% (W/V) Na2HPO4 (BioBasic Inc., Markham, Ontario, Canada), 0.02% (W/V) K2HPO4 (BioBasic Inc., Markham, Ontario, Canada)], the fibroblasts were deprived of serum [DMEM (Dulbecco-Vogt modification of Eagle's medium) (Invitrogen, Burlington, ON, Canada) supplemented with 100 IU/ml penicillin G (Sigma, Oakville, QC, Canada) and 25 μg/ml gentamicin (Schering, Pointe-Claire, QC, Canada) in 5% CO_2_ at 37°C]. This technique has been previously shown to induce secretion of proteins (Villarreal et al., 2013). Using this approach, previous work also demonstrated that short serum deprivation periods did not affect cellular viability from 48 h and inward (Del Galdo et al., 2010; Villarreal et al., 2013; Rogers et al., 2014). Fibroblast's supernatants were therefore collected at different confluence levels and at different collection times after serum deprivation; 24 h pre-confluence, 48 h pre-confluence, 24 h post-confluence, and 48-post confluence (please note that the term pre-confluence is defined as a confluence of 40% and post-confluence as a confluence of 95%). Four tissue culture dishes (75 cm^2^) were seeded for each condition and all the supernatants were collected. They were then centrifuged at 300 g for 10 min (4°C) and kept at −80°C until use. The proteins present in the patient-derived fibroblast conditioned-media were concentrated by the TCA-DOC and the TCA-NLS-THF precipitation methods as described in the Supplementary Data. The protein recovery yield for each culture condition was assessed via a Bradford assay (BioBasic Inc., Markham, Ontario, Canada).

#### TCA-DOC

The supernatants (40 ml) were thawed on ice in high-speed centrifuge tube. 1% (v/v) of a 2% sodium deoxycholate solution (Fisher Scientific, Ottawa, Ontario, Canada) was added to each tube and they were incubated on ice for 30 min after mixing. Trichloroacetic acid was then added to a final concentration of 7.5% (v/v) (BioBasic Inc., Markham, Ontario, Canada) and incubated again on ice for 60 min after mixing. Proteins were precipitated by centrifugation (15,000 g for 20 min at 4°C) and the supernatants were discarded. Twenty milliliters of 100% ice-cold (−20°C) acetone (EMD, Darmstadt, Germany) were added to the pellets, gently vortexed and kept at −20°C for 5 min. After centrifugation (15,000 g for 5 min at 4°C), the supernatants were discarded and 5 ml of 100% ice-cold (−20°C) acetone were added to the pellets. The pellets were again gently vortexed, kept at −20°C for 5 min, centrifuged (15,000 g for 5 min at 4°C), and the supernatant discarded. The pellets were air-dried in a chemical hood for 30 min, dissolved in 210 μl of Isoelectric Focusing (IEF) buffer, pH 8.5 [7M urea (BioBasic Inc., Markham, Ontario, Canada), 2M thiourea (GE Healthcare, Little Chalfont, Buckinghamshire, United Kingdom), 30 mM TRIS (BioBasic Inc., Markham, Ontario, Canada), and 4% CHAPS (BioBasic Inc., Markham, Ontario, Canada)], vortexed, centrifuged (15,000 g for 10 min at room temperature), and kept at −80°C until use.

#### TCA-NLS-THF

The supernatants (40 ml) were thawed on ice in high-speed centrifuge tubes. 1% (v/v) of a 2% sodium layroyl sarcosine solution (Sigma-Aldrich, Oakville, Ontario, Canada) was added to the tubes and they were incubated on ice for 30 min after mixing. Trichloroacetic acid was added to a final concentration of 7.5% (v/v) (BioBasic Inc., Markham, Ontario, Canada) and the supernatants were incubated on ice for 60 min after mixing. Proteins were precipitated by centrifugation (15,000 g for 20 min at 4°C) and the supernatants were then discarded. 10% of the initial volume of ice-cold (−20°C) tetrahydrofuran (BioBasic Inc., Markham, Ontario, Canada) was added, vortexed until complete dissolution of the pellets and kept at −20°C for 5 min. After a centrifugation (15,000 g for 20 min at 4°C), the supernatants were discarded and 10% of the final volume of ice-cold (−20°C) tetrahydrofuran (BioBasic Inc., Markham, Ontario, Canada) was again added, vortexed until complete dissolution of the pellets and kept at −20°C for 5 min. After centrifugation (15,000 g for 20 min at 4°C), the supernatants were discarded and the pellets air-dried in a chemical hood for 30 min, dissolved in 210 μl of IEF buffer (pH 8.5), vortexed, centrifuged (15,000 g for 10 min at room temperature), and kept at −80°C until use.

### Protein conjugation

The proteins were conjugated with fluorescent cyanine dyes (CyDye) and resolved by SDS-PAGE and 2D-DIGE according to standard protocols. The CyDyes were synthesized by the Sherbrooke Molecular Imaging Center based on a protocol by Jung et al. (Jung et al., 2012). Briefly, the internal standard was a mix of equivalent fractions of all the samples used in this study, at a concentration higher than 25 μg, and conjugated with the CyDye Fluor Cy2 dye. The different CyDyes were diluted to a concentration of 400 pmol/μL in dimethylformamide (DMF; BioBasic Inc., Markham, Ontario, Canada). The samples and internal standards were adjusted to a pH of 8.5 with 2M NaOH and conjugated as follow: 30 μg of protein at 1 μg/μL were mixed with 1 μl of each CyDye (400 pmol/μl), vortexed, centrifuged (12,000 g for 30 s at room temperature), and kept on ice for 30 min in the dark. The reaction was stopped with 1 μl of 10 mM L-lysine (BioBasic Inc., Markham, Ontario, Canada), vortexed, centrifuged (12,000 g for 30 s at room temperature), incubated on ice for 10 min, and immediately used or kept at −80°C up to 3 months in the dark.

### Electrophoresis

#### 1D electrophoresis

Gel electrophoresis of the conjugated proteins was performed on a 4% stacking gel and 14% acrylamide/bisacrylamide (37.5:1) resolving gel. Gels were 1.5 mm thick and casted following a specific protocol (Table 2). The migration was done using the Mini-PROTEAN® system (Biorad, Hercules, CA, USA) and performed at a constant rate of 60 mA, 2 h in 1X running buffer (Table 2). Gel readings were done using a Typhoon Trio+ (GE Healthcare, Little Chalfont, Buckinghamshire, United Kingdom). The detection of CyDyes 3 and 5 was done at the optimal photomultiplier voltage (PMT) for each one of them, for each gel analyzed.

**Table 2:** **Detailed protocol for gel and electrophoresis experiments**.

	**Stacking gel**	**Resolving gel**	**10X Running Buffer**	**Light gel**	**Heavy gel**	**Moving solution**	**Conservation buffer**	**DTT solution**	**Iodoacetamine solution**	**Equilibration buffer**	**5X running buffer**	**Overlays solution**
40% acrylamide/bisacrylamide (37.5:1) (w/v)	1 mL	6.3 mL										
1.5M TRIS-HCl, pH 8.8		4.5 L		52.5 mL	52.5 mL	50 mL	100 mL			50 mL		
0.5M TRIS-HCl, pH 6.8	2.5 mL											
10% SDS (w/v)	0.1 mL	0.18 mL		2.1 mL	2.1 mL		4 mL					
10% APS	0.05 mL	0.09 mL		2.1 mL	1.05 mL							
TEMED	0.015 mL	0.009 mL		360 μL	60 μL							
Glycine			120 g									
SDS			40 g							20 g	40 g	
TRIS			576 g								60.5 g	
30% acrylamide/bisacrylamide (37.5:1) (w/v)				68 mL	128 mL							
Glycerol					18 mL	74 g				378 g		
1% bromophenol blue					210 μL							20 μL
1M DTT								0.1 g				
Iodoacetamine									1.12 g			
Equilibration buffer								10 mL	25 mL			
Urea										355 g		
Agarose type I-A												0.4 g
Agarose type VII												0.1 g
5X running buffer												20 mL
Apyrogenic water	6.35 mL	6.93 mL	to 4L	85 mL	14 mL	90 mL	296 mL			To 1L	to 4L	80 mL

*Acrylamide/bisacrylamide (37.5:1) solution (w/v) (BioBasic Inc, Markham, Ontario, Canada), agarose type 1-A (Sigma, Oakville, Québec, Canada), ammonium persulfate (BioBasic Inc, Markham, Ontario, Canada), bromophenol blue (Sigma, Oakville, QC, Canada), dithiothreitol (DIT) (BioBasic Inc, Markham, Ontario, Canada), glycerol (BioBasic Inc, Markham, Ontario, Canada), glycine (BioBasic Inc, Markham, Ontario, Canada), iodoacetamide (BioBasic Inc, Markham, Ontario, Canada), sodium dodecyl sulfate (SDS) (BioBasic Inc, Markham, Ontario, Canada), TRIS (BioBasic Inc, Markham, Ontario, Canada), TRIS-HCI (BioBasic Inc, Markham, Ontario, Canada), tetramethylethylenediamine (TEMED) (BioBasic Inc, Markham, Ontario, Canada), urea (BioBasic Inc, Markham, Ontario, Canada), 2-hydroxythylagarose type VII (Sigma, Oakville, Quebec, Canada)*.

#### 2D electrophoresis

The different CyDyes mixes were pooled together. Ninety microliters of the CyDye mix was added to 90 μl of a reduction solution [7M urea (BioBasic Inc., Markham, Ontario, Canada), 2M thiourea (GE Healthcare, Little Chalfont, Buckinghamshire, United Kingdom), 4% (w/v) CHAPS (BioBasic Inc., Markham, Ontario, Canada), 0.5% (v/v) IPG (GE Healthcare, Little Chalfont, Buckinghamshire, United Kingdom), 1 mM dithiothreitol (GE Healthcare, Little Chalfont, Buckinghamshire, United Kingdom), 0.05% (v/v) bromophenol blue (Sigma, Oakville, QC, Canada)], vortexed and incubated for 15 min on ice in the dark. The sample was centrifuged (21,000 g for 30 s at room temperature) and the supernatant collected. The dyes were randomly conjugated to the different protein samples.

The first dimension of the 2D electrophoresis, the isoelectric focusing, was done on Immobiline DryStrip pH 3-11 NL, 24 cm (GE Healthcare, Little Chalfont, Buckinghamshire, United Kingdom) strips. They were rehydrated at room temperature overnight with 450 μl of a mix of the remaining reduced supernatant and rehydration solution [7M urea (BioBasic Inc., Markham, Ontario, Canada), 2M thiourea (GE Healthcare, Little Chalfont, Buckinghamshire, United Kingdom), 4% CHAPS (BioBasic Inc., Markham, Ontario, Canada), 0.5% IPG (GE Healthcare, Little Chalfont, Buckinghamshire, United Kingdom), 1.2% DeStreak rehydration solution (GE Healthcare, Little Chalfont, Buckinghamshire, United Kingdom), 0.05% bromophenol blue (Sigma, Oakville, QC, Canada)]. Migration of the strips was done with the Ettan IPGphor 3 IEF system (GE Healthcare, Little Chalfont, Buckinghamshire, United Kingdom) following a specific protocol (Table 3). The strips were covered with Plus One DryStrip cover fluid (GE Healthcare, Little Chalfont, Buckinghamshire, United Kingdom) during the entire migration and kept at −80°C until use.

**Table 3:** **Detailed migration protocol for the 2D gel electrophoresis**.

	**U (V)**	**Time or Vh**	**μA (I/strip)**	**Temperature (°C)**
Step 1	500	500	75	20
Step 2	1000	800	75	20
Step 3	10,000	16,500	75	20
Step 4	10,000	32,200	75	20

The second dimension of the 2D electrophoresis was done on 10–18% gradient polyacrylamide gels. The gels (26 × 20 cm) were casted with the DALT*six* gel caster and the DALT*six* gradient maker (GE Healthcare, Little Chalfont, Buckinghamshire, United Kingdom). The gels were cast following a specific recipe (Table 2) combining light and heavy acrylamide solutions, in order to generate a linear gradient between the two concentrations. The light gel represents the solution with the lowest percentage of total acrylamide (9.7%) while the heavy gel represents the solution with the highest (maximum) percentage of total acrylamide (17.8%). The gels were covered with 30% isopropyl alcohol (Laboratoire MAT, Québec, Québec, Canada) and polymerized for 3 h. The gels were then washed with apyrogenic water, covered with a conservation buffer (Table 2) and kept polymerizing for another 2 h.

The strips were thawed at room temperature and washed for 20 min under agitation in a DTT solution (Table 2). A second 20 min wash under agitation in an iodoacetamide solution (Table 2) was done and the strips were added on top of the gels. To maintain them in place, an overlays solution was added over the strips (Table 2).

The migration was done using the Ettan DALT*six* large vertical system and performed at a constant rate of 0.5 W/gel for 1 h and then over night at a constant rate of 1.25 W/gel in 1X running buffer (Table 2). The whole system was kept at 20°C with a water-cooling system. Gels readings were done using a Typhoon Trio+ (GE Healthcare, Little Chalfont, Buckinghamshire, United Kingdom) as described above.

### Visualization and quantification of the in-gel fluorescent signals

CyDye-labeled gels were visualized using a Typhoon Trio Plus laser scanner (GE Healthcare). Excitation and emission wavelengths were chosen according to manufacturer's recommendations (488, 532, and 633 nm laser beams; 520, 580, and 670 nm emission filters). Quantification of the fluorescent signal was done using ImageJ software. The average pixel saturation of the fluorescent signal from the Typhoon Image was calculated and normalized for background fluorescence.

## Results

### Comparison of protein extraction methods and the effect of the confluence level and collection time on the protein recovery yield

**Figure 1:**
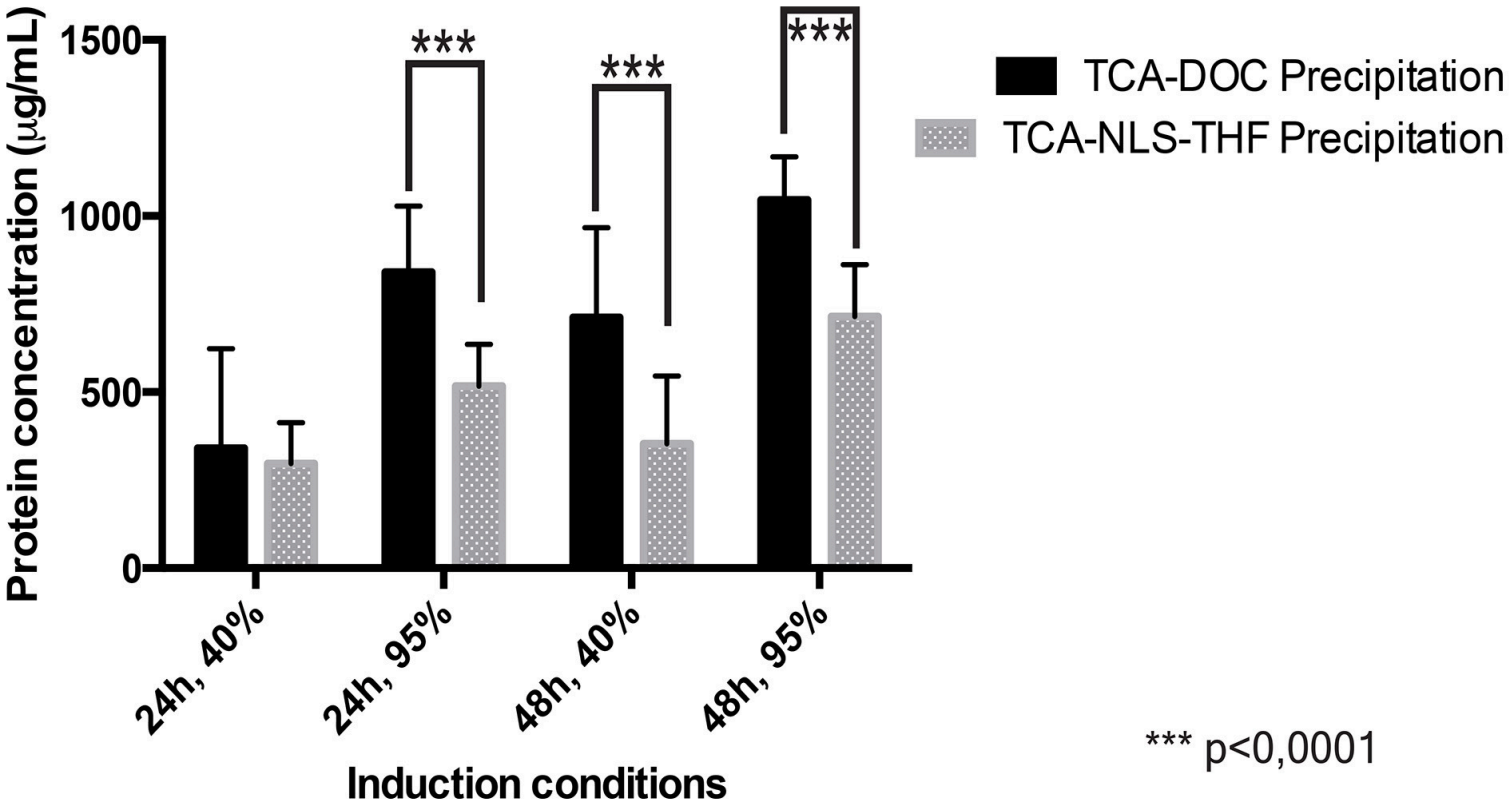
**Protein recovery yield in function of precipitation methods, collection times, and confluence levels measured by the Bradford assay**. The yield of protein recovered following the two tested protein precipitation methods (TCA-DOC and TCA-NLS-THF) and four different culture conditions was measured by the Bradford assay. The amount of recovered protein for all the tested conditions is given in μg/ml of conditioned-supernatant. The TCA-DOC method consistently gave better recovery yields independent of the induction conditions that were used. ^***^Corresponds to a *P*-value of < 0.0001.

Repeated experiments, using fibroblasts collected from two different neuronal pathologies (SALS/FALS and neurofibromatosis) and controls revealed that the TCA-DOC protein precipitation method was consistently superior to the TCA-NLS-THF method to recover proteins (Figure 1). A statistically significant difference between the two used protein precipitation methods was revealed by a grouped analysis. A 2-way ANOVA followed by a Bonferroni *post-hoc* test with a significance level of 0.05 was done using PRISM software (GraphPad Software, Inc., San Diego). The error bar represents the standard deviation. The TCA-DOC protein precipitation method was consistently giving higher recovery yield when compared to the TCA-NLS-THF method for each tested condition (Figure 1). No significant differences were observed in the recovered amount of secreted proteins between ALS (SALS/FALS), NF1, and controls fibroblasts. We then tested the influence of the confluence level as well as the collection time (24 and 48 h at 40 or 95% of confluence) on the total amount of secreted proteins recovered from each fibroblast conditioned-media. The 48 h post-confluency culture condition for both protein precipitation methods was proven to give the better recovery yield (Figure 1). However, the higher recovery yield was consistently obtained after a 48 h induction at 95% of cell confluence with the TCA-DOC (1045.2 μg/ml) protein precipitation method compared to the TCA-NLS-THF method (713.9 μg/ml).

### Effect of cyanine dye conjugation on the recovered protein

**Figure 2:**
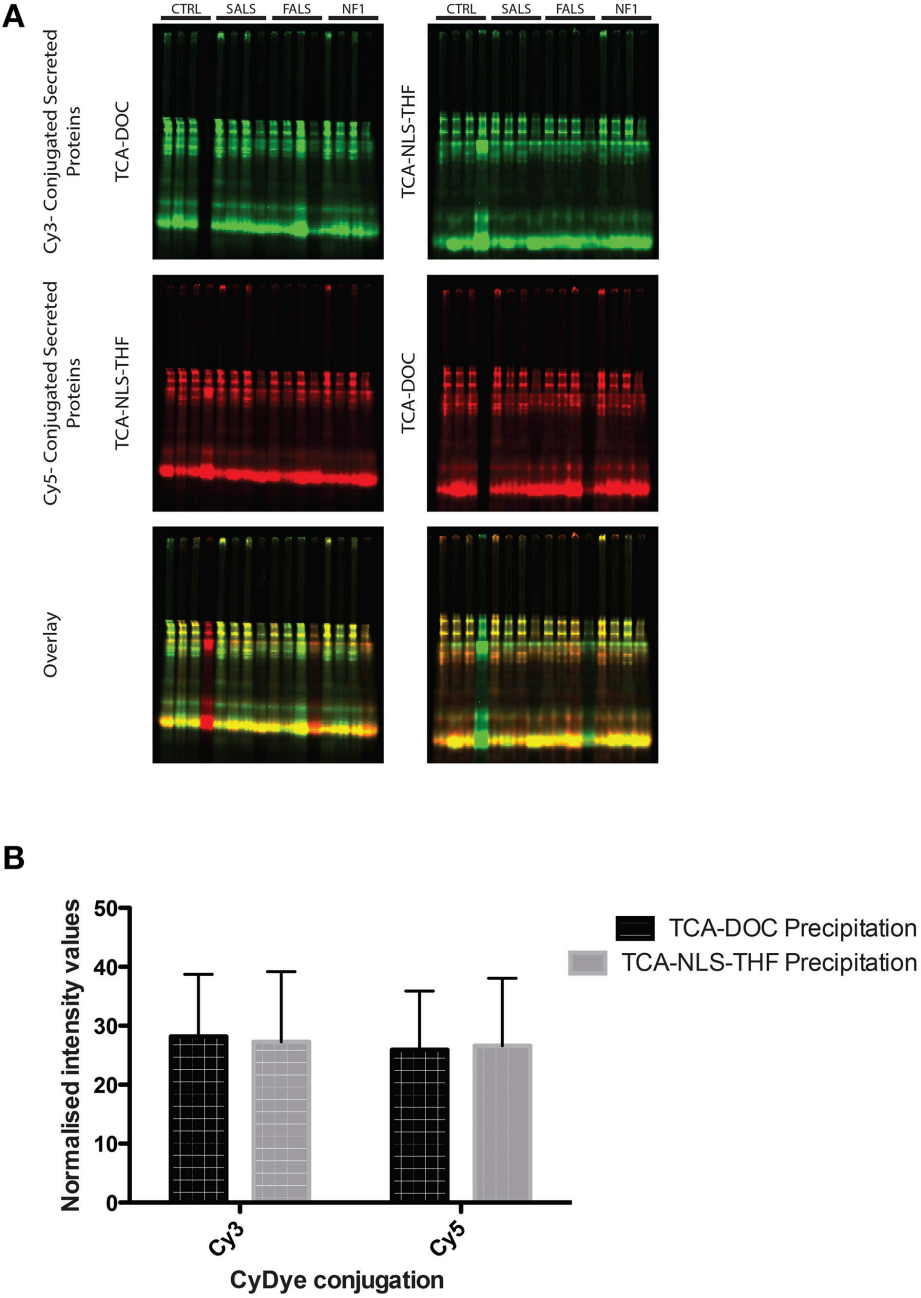
**One dimension SDS-PAGE gel of secreted proteins precipitated from fibroblast conditioned-medium and conjugated with CyDyes. (A)** Conjugation of CyDyes 3 and 5 to TCA-DOC and TCA-NLS-THF precipitated proteins. A 14% acrylamide/bisacrylamide (37.5:1) gel was used to resolve the CyDye conjugated supernatant protein. An internal standard was produced by conjugating protein from the TCA-DOC (Post-48 h) with both Cy3 (green) and Cy5 (red) dyes. One fibroblast cell line per tested condition for each of the studied pathology and control was tested and loaded in the gels in this order: pre-confluence/24 h, post-confluence/48 h, pre-confluence/48 h, post-confluence/48 h. Equal amount of proteins was loaded in all the gels. **(B)** Quantification of the fluorescent signal, detected with our laser-scanner Typhoon device, was done using ImageJ software. No statistical differences were noted for each of the tested condition, indicating that any of the protein precipitation method used in this study did not interfere with the CyDye conjugation.

In order to determine if any of the protein precipitation methods interfered with cyanine dye conjugation, we performed a one dimension SDS-PAGE gel of secreted proteins precipitated from fibroblast conditioned-medium and conjugated with different commonly used CyDyes. As illustrated in Figure 2, both the TCA-DOC and TCA-NLS-THF methods have no significant effect on the conjugation with the CyDyes (3 and 5; Figure 2). A 2-way ANOVA followed by a Bonferroni *post-hoc* test with a significance level of 0.05 was done using PRISM software (GraphPad Software, Inc., San Diego). The error bar represents the standard deviation. The TCA-DOC method was therefore selected for downstream 2D-DIGE proteomic studies since it gives the higher and significant (*P* < 0.0001) recovery yield for all the induction conditions tested.

### Visualization of whole secreted protein resolved with 2D-DIGE

**Figure 3:**
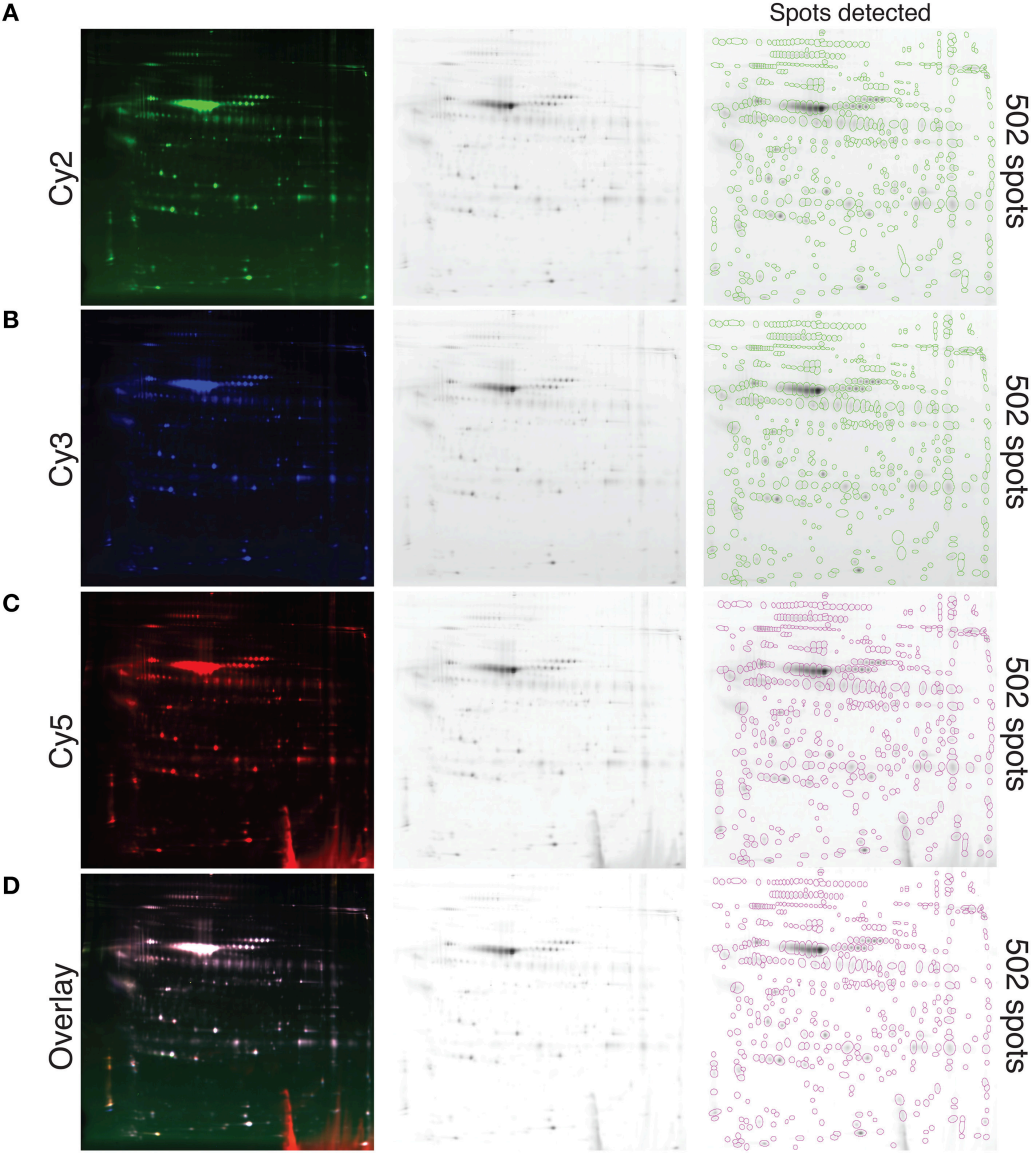
**Visualization of whole secreted protein and quantification of the protein spots resolved with 2D-DIGE at the optimal induction condition**. Conjugation of each CyDye, Cy2 **(A)**, Cy3 **(B)**, and Cy5 **(C)**, to precipitated proteins extracted from fibroblast conditioned-medium via the TCA-DOC method and resolved by 2D-DIGE is shown. Please note that all technical replicates (disease and control) obtained for the 48 h post-confluence induction condition were pooled together prior CyDye conjugation and loaded on gel. **(D)** Represents a fusion image of the three different CyDyes. Each individual spot is circled. The number of detected spots (502) is indicated at the far right. Equal number of spots is detected for each of the tested CyDyes indicating that each of the specific tested CyDyes bond with the same effectiveness and therefore that the conjugation efficiency is independent of the CyDye fluors.

**Figure 4:**
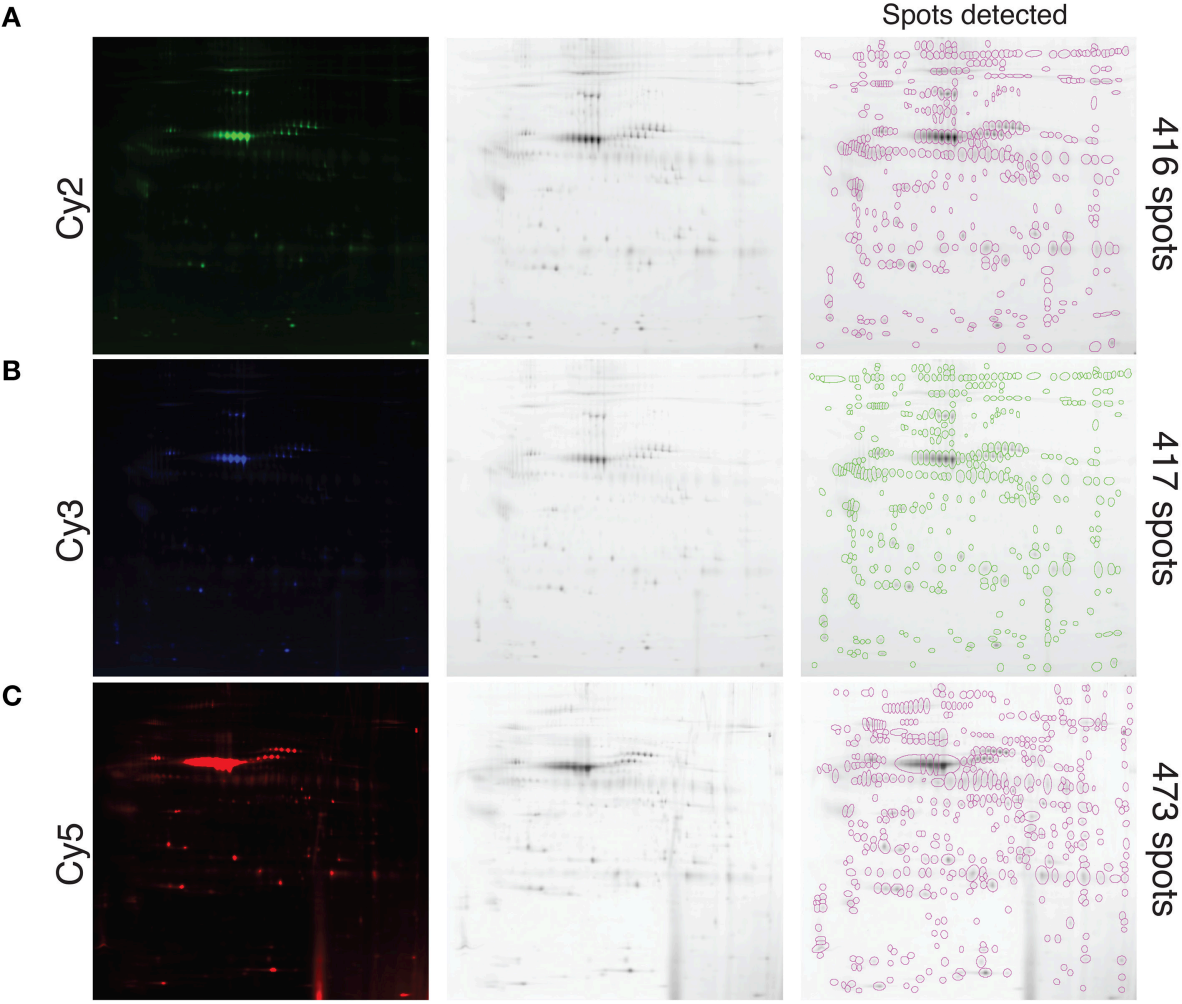
**Visualization and quantification of the protein spots of different tested induction conditions and precipitation methods**. Proteins were conjugated with the different CyDyes after extraction with the TCA-DOC, 24 h, post-confluence (Cy2) **(A)**, TCA-NLS-THF, 48 h, post-confluence (Cy3) **(B)** and TCA-DOC, 48 h post-confluence (Cy5) **(C)** induction conditions and precipitation methods and resolved by 2D-DIGE from one control cell line. Each individual spot is circled and was automatically quantified by the software (far right). Note that the TCA-DOC 48 h post-confluence induction condition, which was also consistently given better protein recovery yield, gave the highest number of protein spots.

A pool of precipitated proteins collected from all the samples tested in this study, using the TCA-DOC method 48 h post-confluence and induction, were conjugated with each CyDyes (2, 3, and 5) and resolved with 2D-DIGE. The results show that each CyDyes bind at the same effectiveness to the proteins, giving an equal number of detected spots (502) for each of the conjugation (Figure 3) indicating that the choice of a specific CyDye does not affect or interfere with protein detection. Conjugation of the samples for each tested conditions was done in a randomized fashion between the two CyDyes used in the study (Cy3 and Cy5). The conjugated proteins mixture, precipitated from fibroblast conditioned-medium for two different conditions, was subjected to isoelectric focusing followed by a 10–18% SDS-PAGE gradient migration in the 2nd dimension. To determine the number of spots detected, the Delta 2D analysis software V4.2 (Decodon, Greifswald, Germany) was used. A data pool, comprised of all the gels and the CyDyes, was created. Quantification of the number of spots detected between different induction conditions and precipitation methods (TCA-DOC post-confluence, 24 h of incubation/TCA-DOC, post-confluence, 48 h of incubation/TCA-NLS-THF, post-confluence, 48 h of incubation) was also assessed. In line with our previous results showing that the TCA-DOC 48 h post-confluency was given better protein recovery yield, more spots were also detected using this method when compared to the two other conditions tested (Figure 4).

## Discussion

The proteins secreted by a particular type of cell, the secretome, play important roles in the regulation of many physiological processes via paracrine/autocrine mechanisms, and they are of increasing interest as potential biomarkers and therapeutic targets in diseases (Pocsfalvi et al., 2011). The proteins released by cells into conditioned media *in vitro* have been studied to better understand pathological conditions and mechanisms *in vivo* (Pocsfalvi et al., 2011; Ribeiro et al., 2011). However, the secretome analysis still faces some problem mainly related to cell preparation, protein extraction, enrichment, and conjugation. To facilitate ongoing research involving secreted proteins, we revisited cell culture protocols and whole secreted protein enrichment. We identified the optimal fibroblast cells culture protocol, secreted protein collection, as well as precipitation method for the subsequent secretomic screening experiments. The maximum recovery yield was obtained when collecting the conditioned supernatants 48 h after induction when fibroblasts were at 95% of confluence with the TCA-DOC precipitation method. Washing the cells three times with PBS before serum deprivation ruled out possible contaminants present in the bovine serum (Rogers et al., 2014). It cannot be excluded however that some intracellular proteins, released in the medium by cellular lysis, might be present in the collected supernatants. Nonetheless, since the serum-deprived protocol used to extract proteins was the same for all tested conditions, it is unlikely that the presence of lysis-derived contaminants would interfere with the analysis and give false-positive results. Moreover, it has been previously shown that the removal of bovine protein, especially when using PBS, seems not to compromise the number of living cells and to minimize the effects on cell lysis and viability (Rogers et al., 2014). A comparative approach between the conditioned-medium and cytosolic extract should however be performed in order to normalize the results for the presence of minimized lysis-derived contaminants. The concept of DIGE was originally developed by Unlü and colleagues (Unlü et al., 1997). This technique allows higher reproducibility and provides information about more subtle changes in protein expression than conventional 2D electrophoresis as three different sample types can be run on the same gel and be directly compared to one another, one being the internal standard (i.e., a pool of all the samples of the experiment). This technique, relying on pre-electrophoretic labeling of samples with one of three spectrally resolvable fluorescent cyanine dyes (Cy2, Cy3, and Cy5), also allows multiplexing of samples into the same gel, and has become the method of choice over the last years for comparative proteomic profiling in a cost-effective manner. With the different 2-D gel image analysis software available commercially, spot detection as well as statistical analysis, proteome maps and spot picking, for mass spectrometry (MS) analysis, can easily be done. Table 4 presents a list of available software. Therefore, we have identified the optimal technical parameters that shall be used in the subsequent 2D fibroblast secretome profiling experiments. According to our results, we therefore propose that protein precipitation should be performed by TCA-DOC precipitation method after 48 h at 95% of confluence in a serum-deprived culture medium.

**Table 4:** **Available 2D gel analysis software and spot quantification**.

**Company**	**Software**
Bio-Rad, Hercules, CA, USA	PDQuest-2D
DECODON, Greifswald, Germany	Delta2D
GE Healthcare, Little Chalfont, Buckinghamshire, United Kingdom	DeCyder 2D/Image Master 2D Platinum
Genbio, Geneva, Switzerland	Melanie
Ludesi, http://www.ludesi.com	Redfin
Syngene, Frederick, MD, USA	Dymension
Totallab, Newcastle upon Tyne, United Kingdom	SameSpots

Comparative proteomic profiling is a relevant approach to biomarker discovery and is becoming extensively utilized in secretome analysis. Mainly, two prominent sources of secreted proteins have been utilized in secretomic studies: cell line supernatants derived from patients and proximal biological fluids (blood, sera, cerebral spinal fluid, etc.). The availability of skin fibroblasts is particularly advantageous for research involving neurological diseases, since it is difficult to obtain affected tissues from living patients. The main function of fibroblasts is to maintain the structural integrity of connective tissues by continuously secreting precursors of the extracellular matrix. Fibroblasts are readily cultured in research laboratories and incorporation of fibroblasts into tissue-engineered skin substitutes has produced encouraging results in the treatment of severely burned patients, symptomatic pain relief, more rapid healing of acute, chronic wounds, and even to model different diseases such as ALS and psoriasis (Auger et al., 1998, 2009; Moulin et al., 2000; Auxenfans et al., 2009; Jean et al., 2009; Paré et al., 2015). As a matter of fact, this novel tissue-engineered skin model derived from both sporadic and familial ALS patients was reported to be the first to present cytoplasmic TDP-43 accumulation in cells outside of the nervous system. Furthermore, generation of human induced pluripotent stem cells (iPSCs) from fibroblast cells provides a new method to elucidate the molecular basis of human diseases and a novel approach to disease modeling and drug discovery (Takahashi et al., 2007; Yu et al., 2007). Actually, an increasing number of studies have employed disease-specific human iPSCs to model neurological diseases, including Parkinson's disease, Autism spectrum disorders, Friedreich's Ataxia, spinal muscular atrophy, ALS, Angelman syndrome and Prader-Willi syndrome, and only few of them have demonstrated disease-specific phenotypes (Dimos et al., 2008; Park et al., 2008; Ebert et al., 2009; Soldner et al., 2009; Chamberlain et al., 2010; Ku et al., 2010; Marchetto et al., 2010; Nguyen et al., 2011; Ryan et al., 2013; Velasco et al., 2014). The potential of iPSCs technology is enormous, but major obstacles to iPSC studies for late-onset neurodegenerative diseases remains. Their production is still very expensive and time consuming. iPSC also show genetic and epigenetic abnormalities after time in culture, sometimes even showing cancer-related mutations (Gore et al., 2011; Bhartiya et al., 2013). This present study can overcome these mutations-related problems since the skin fibroblasts are used without any genetic modifications. They are also easier to work with than iPSC-derived neurons since the culture medium needed is less expensive and doesn't need to be supplemented of various chemical and biological compounds such as nerve growth factors. The identical germ layer origin of both the skin and neural tissue leads us to think that skin can also be used to study neurodegenerative diseases. For instance, cytoplasmic accumulation of TDP-43, a pathological signature of ALS and ALS/FTD, was observed in the skin of ALS patients and in ALS-derived tissue engineered skins (Paré et al., 2015). Thus, fibroblast cells, able to secrete extracellular matrix molecules, as the matrix metalloproteinases (MMPs), which were shown to be associated with neural morphogenesis as well as being important in the synaptic remodeling, on both the structural and functional levels (McFarlane et al., 2003; Rivera et al., 2010; Huntley, 2012), could be of major importance in understanding pathophysiological mechanisms of neurodegenerative diseases.

## Conclusion

Comparative proteomic profiling of patient-derived fibroblasts to elucidate the pathogenesis of rare diseases, especially neurological disorders, presents an attractive alternative to neural tissue biopsies from living patients that are of limited access. These new techniques will hopefully help researchers and clinicians to better understand these yet untreatable diseases. Conceivably, the proposed approach based on the study of patient-derived skin fibroblasts to facilitate the identification of biomarkers for early diagnosis or to monitor disease progression becomes highly attractive and of high importance in clinical neurology. A comprehensive survey of patient-derived whole secreted proteins in fibroblast-conditioned media will certainly lead to more translational research to facilitate the identification of specific disease biomarkers and new molecular targets for therapy or molecular signatures to improve early diagnosis, to find a cure or to slow down the course of this yet untreatable diseases.

## Author contributions

Experimental and technical inputs: BP, LD. Study design: BP, RP, and FGL. Recruitment and clinical assessement of patients: ND. Writing of the article: BP, RP, ND, and FGL. Operational grant money: FGL.

## Funding

This work was supported by ALS Canada, the Canadian Institutes for Health Research and the W. Garfield Weston Foundation through the Weston Brain Institute. Part of this work was also supported by the Fondation des Hôpitaux Enfant-Jésus—St-Sacrement.

### Conflict of interest statement

The authors declare that the research was conducted in the absence of any commercial or financial relationships that could be construed as a potential conflict of interest.
