# Erratum: An Optimized Approach to Recover Secreted Proteins from Fibroblast Conditioned-Media for Secretomic Analysis

**DOI:** 10.3389/fncel.2016.00107

**Published:** 2016-04-25

**Authors:** Frontiers Production Office

**Affiliations:** Frontiers Production Office, FrontiersSwitzerland

**Keywords:** secretomic, 2D-DIGE, secreted proteins, secretome, fibrolast-conditioned media

Reason for Erratum:

Due to a typesetting error, a misalignment in Table 2 lead to the publication of incorrect information. In the “Resolving gel” column, line “1.5M TRIS-HCl, pH 8.8,” the volume should be 4.5 mL, and not 4.5 L as published.

**Table 2:** **Detailed protocol for gel and electrophoresis experiments**.

	**Stacking gel**	**Resolving gel**	**10X Running Buffer**	**Light gel**	**Heavy gel**	**Moving solution**	**Conservation buffer**	**DTT solution**	**Iodoacetamine solution**	**Equilibration buffer**	**5X running buffer**	**Overlays solution**
40% acrylamide/bisac rylamide (37.5:1) (w/v)	1 mL	6.3 mL										
1.5M TRIS-HCl, pH 8.8		4.5 mL		52.5 mL	52.5 mL	50 mL	100 mL			50 mL		
0.5M TRIS-HCl, pH 6.8	2.5 mL											
10% SDS (w/v)	0.1 mL	0.18 mL		2.1 mL	2.1 mL		4 mL					
10% APS	0.05 mL	0.09 mL		2.1 mL	1.05 mL							
TEMED	0.015 mL	0.009 mL		360 μL	60 μL							
Glycine			120 g									
SDS			40 g							20 g	40 g	
TRIS			576 g								60.5 g	
30% acrylamide/bisac rylamide (37.5:1) (w/v)				68 mL	128 mL							
Glycerol					18 mL	74 g				378 g		
1% bromophenol blue					210 μL							20 μL
1M DTT								0.1 g				
Iodoacetamine									1.12 g			
Equilibration buffer								10 mL	25 mL			
Urea										355 g		
Agarose type I-A												0.4 g
Agarose type VII												0.1 g
5X running buffer												20 mL
Apyrogenic water	6.35 mL	6.93 mL	to 4L	85 mL	14 mL	90 mL	296 mL			To 1L	to 4L	80 mL

Acrylamide/bisacrylamide (37.5 :1) solution (w/v) (BioBasic Inc, Markham, Ontario, Canada), agarose type I-A (Sigma, Oakville, Québec, Canada), ammonium persulfate (BioBasic Inc, Markham, Ontario, Canada), bromophenol blue (Sigma, Oakville, QC, Canada), dithiothreitol (DTT) (BioBasic Inc, Markham, Ontario, Canada), glycerol (BioBasic Inc, Markham, Ontario, Canada), glycine (BioBasic Inc, Markham, Ontario, Canada), iodoacetamide (BioBasic Inc, Markham, Ontario, Canada), sodium dodecyl sulfate (SDS) (BioBasic Inc, Markham, Ontario, Canada), TRIS (BioBasic Inc, Markham, Ontario, Canada), TRIS-HCl (BioBasic Inc, Markham, Ontario, Canada), tetramethylethylenediamine (TEMED) (BioBasic Inc, Markham, Ontario, Canada), urea (BioBasic Inc, Markham, Ontario, Canada), 2- hydroxythylagarose type VII (Sigma, Oakville, Québec, Canada).

The publisher apologizes for this error and the correct version of Table 2 appears below.

This error does not change the scientific conclusions of the article in any way.

