# Supplementary material for: An Optimized Approach to Recover Secreted Proteins from Fibroblast Conditioned-Media for Secretomic Analysis
Source: Front Cell Neurosci. 2016 Mar 31;10:70. doi: 10.3389/fncel.2016.00070 (PMC4814560; doi:10.3389/fncel.2016.00070)
Supplement: Supplementary file 1 [file DataSheet1.pdf]

Supplementary data – Protocol

An optimized approach to recover secreted proteins from fibroblast conditioned-media for secretomic analysis

Bastien Paré[1,4], Lydia Touzel Deschênes[1], Roxane Pouliot[1,2] Nicolas Dupré[3] and Francois Gros-Louis[1, 4, *]

1. Division of Regenerative Medicine, Laval University Experimental Organogenesis Research Center/LOEX, CHU de Québec Research Center – Enfant-Jésus Hospital, Québec, QC, Canada
2. Faculty of Pharmacy, Laval University, Québec, QC, Canada
3. Neuroscience Division of the CHU de Québec, Department of Medicine of the Faculty of Medicine, Laval University, Québec, QC, Canada
4. Department of Surgery, Faculty of Medicine, Laval University, Québec, QC, Canada

* Francois Gros-Louis, PhD
Centre de Recherche en Organonogénèse Expérimentale / LOEX
Centre de Recherche du CHU de Québec – Hôpital de l'Enfant-Jésus
1401, 18e rue
Québec, Canada, G1J 1Z4
Francois.gros-louis@fmed.ulaval.ca

- Fibroblast cells extraction
  - Incubate the biopsies in 0.05% thermolysin overnight at 4°C.
  - Separate the epidermis from the dermis mechanically.
  - Isolate fibroblasts from the dermis after treatment with 0,2 IU/ml collagenase H.

- Fibroblast cells culture
  - Grow cells in DMEM (Dulbecco-Vogt 133 modification of Eagle's medium) supplemented with 10% Bovine Growth Serum (HyClone BGS), 100 IU/ml penicillin G and 25 μg/ml gentamicin in 5% CO_2 at 37°C from a concentration of 2,5 x 10^5 cells on tissue culture dishes (75 cm^2) to desired confluence (40 or 95% of confluence).

- Fibroblasts induction
  - Wash 3 times with 10 ml of phosphate-buffered saline (PBS).
  - Deprive fibroblasts of serum (DMEM supplemented with 100 IU/ml penicillin G and 25 μg/ml gentamicin).
  - Incubate for the desired time (24h or 48h) in 5% CO_2 at 37°C.
  - Collect the supernatants and centrifuge at 300g for 10 minutes (4°C). Keep at -80°C until use.

- TCA-DOC precipitation
  - Thaw on ice the supernatants in high-speed centrifuge tube.
  - Add 1% (v/v) of a 2% sodium deoxycholate solution to the supernatants and incubate on ice for 30 minutes after mixing.
  - Add 100% trichloroacetic acid to a final concentration of 7,5% (v/v) and incubate on ice for 60 minutes after mixing.
  - Precipitate the proteins by centrifugation (15,000g for 20 minutes at 4°C) and discard the supernatants.
  - Add 20 ml of 100% ice-cold (-20°C) acetone to the pellets, vortex gently and keep at -20°C for 5 minutes.
  - Centrifuge (15,000g for 5 minutes at 4°C) and discard the supernatants.
  - Add 5 ml of 100% ice-cold (-20°C) acetone, vortex gently and keep at -20°C for 5 minutes.
  - Centrifuge (15,000g for 5 minutes at 4°C) and discard the supernatants.
  - Air-dry the pellets in a chemical hood for 30 minutes and dissolve in 210 μl of Isoelectric Focusing (IEF) buffer, pH 8.5.
  - Vortex the samples, centrifuge at 15,000g for 10 minutes at room temperature and keep at -80°C until use.

- TCA-NLS-THF precipitation
  - Thaw on ice the supernatants in high-speed centrifuge tube.
  - Add 1% (v/v) of a 2% sodium layroyl sarcosine solution to the supernatants and incubate on ice for 30 minutes after mixing.
  - Add 100% trichloroacetic acid to a final concentration of 7,5% (v/v) and incubate on ice for 60 minutes after mixing.

- Precipitate the proteins by centrifugation (15,000g for 20 minutes at 4°C) and discard the supernatants.
- Add 10% of final volume of ice-cold (-20°C) tetrahydrofuran to the pellets, vortex until dissolution of the pellets and keep at -20°C for 5 minutes.
- Centrifuge (15,000g for 20 minutes at 4°C) and discard the supernatants.
- Add 10% of final volume of ice-cold (-20°C) tetrahydrofuran to the pellets, vortex until dissolution of the pellets and keep at -20°C for 5 minutes.
- Centrifuge (15,000g for 20 minutes at 4°C) and discard the supernatants.
- Air-dry the pellets in a chemical hood for 30 minutes and dissolve in 210 μl of Isoelectric Focusing (IEF) buffer, pH 8.5.
- Vortex the samples, centrifuge at 15,000g for 10 minutes at room temperature and keep at -80°C until use.

- Protein conjugation
    - Dilute the different CyDyes to a concentration of 400pmol/μl in dimethylformamide (DMF).
    - Adjust the samples to a pH of 8.5 with 2M NaOH.
    - Mix 30 μg of protein at 1 μg/ml with 1 μl of the desired CyDye.
    - Vortex the samples, centrifuge (12,000g for 30 seconds at room temperature) and keep on ice for 30 minutes in the dark.
    - Stop the reaction with 1 μl of 10mM L-lysine.
    - Vortex the samples, centrifuge (12,000g for 30 seconds at room temperature) and keep on ice for 10 minutes in the dark.
    - Immediately use or keep at -80°C for up to 3 months in the dark.

- 2D electrophoresis
    - Pool the conjugated proteins together (Cy2, Cy3 and Cy5).
    - Add 90 μl of the mix to 90μl of reduction solution, vortex and incubate for 15 minutes on ice in the dark (Table 2).
    - Centrifuge (21,000g for 30 seconds at room temperature) and collect the supernatant.

        - First dimension electrophoresis
            - Rehydrate the Immobiline 230 DryStrip pH 3-11 NL, 24 cm at room temperature overnight in 450 μl of a mix of the remaining reduced supernatant and rehydration solution (Table 2).
            - Migrate the strips following the protocol mentioned in table 3.
            - Recover the strips with Plus One DryStrip cover fluid during migration and keep them at -80°C until use.

        - Second dimension electrophoresis
            - Cast 10-18% gradient polyacrylamide gels (26 X 20 cm) with the DALT*six* gel caster and the DALT*six* gradient maker.
            - Cast the gels (light then heavy gel) then the moving solution following the specific recipe (Table 2).

- Cover the gels with 30% isopropyl alcohol and let polymerize for three hours.
- Wash the gels with apyrogenic water, cover them with conservation buffer (Table 2) and let polymerize for another two hours.
- Thaw at room temperature the strips and wash them 20 minutes under agitation in a DTT solution (Table 2).
- Discard the DTT solution and wash the strips 20 minutes under agitation in an iodoacetamide solution (Table 2).
- Discard the iodoacetamide solution and add the strips on top of the gels.
- Add an overlays solution over the strips to maintain them in place (Table 2).
- Migrate the gels with the Ettan DALT*six* large vertical system at a constant rate of 0.5W/gel for 1 hour then over night at a constant rate of 1.25W/gel in 1X running buffer (Table 2).
- Keep the whole system at 20°C with a water-cooling system.

- Gels reading
    - Read the gels with the appropriate reading system at the optimal photomultiplier voltage (PMT) for each one of them, for each gel analyzed.
